# A preliminary review of warfarin toxicity in a tertiary hospital in Cape Town, South Africa

**DOI:** 10.5830/CVJA-2017-029

**Published:** 2017

**Authors:** Annemarie Jacobs,, Eric H Decloedt, Fatima Bassa

**Affiliations:** Faculty of Medicine and Health Sciences, University of Stellenbosch, Cape Town, South Africa; Division of Clinical Pharmacology, Department of Medicine, Faculty of Medicine and Health Sciences, University of Stellenbosch, Cape Town, South Africa; Division of Haematology, Department of Medicine, Faculty of Medicine and Health Sciences, University of Stellenbosch, Cape Town, South Africa

**Keywords:** warfarin, toxicity, bleeding, treatment, cost

## Abstract

**Aim:**

Warfarin is a widely used anticoagulant for the prevention and treatment of thromboembolism. We conducted a retrospective review to determine the causes and management of warfarin toxicity of patients admitted to Tygerberg hospital between June 2014 and June 2015.

**Results:**

We identified and evaluated 126 patients who met the inclusion criteria. The cause of warfarin toxicity was identified and addressed in only 14.3% (18/126) of patients. Where the cause was identified, 56% (10/18) was due to dosing errors and 17% (3/18) drug–drug interaction (DDI). However, 77% (97/126) of patients were retrospectively identified as receiving concomitant medicines known to interact with warfarin at the time of admission. Twenty-eight per cent (35/126) of patients presented with major bleeding, which included seven cases of intracranial haemorrhage. Patients were admitted for a median of eight days at an average treatment cost of R10 578.

**Conclusion:**

We found that warfarin toxicity carries significant mortality and cost, but little attention is paid to the causes of toxicity.

## Introduction

Warfarin is a widely used anticoagulant indicated for the prevention and treatment of thromboembolism in patients with atrial fibrillation, prosthetic heart valves and deep-vein thrombosis. However, warfarin therapy is challenging. Sonuga et al.^1^ reported in a study done at Victoria Hospital, Cape Town, that a therapeutic international normalised ratio (INR) outcome was achieved in only 48.5% of patients. The warfarin dose–response curve is not predictable and requires regular INR monitoring to optimise efficacy and minimise toxicity.^2^

Cytochrome p450 2C9 (CYP2C9) and vitamin K epoxide reductase complex subunit 1 (VKORC1) genetic polymorphisms contribute to clinically significant variability in warfarin exposure and efficacy.^2^,^3^ Genetic polymorphisms in the CYP2C9 and VKORC1 enzymes account for 10 to 15% and 20 to 35% of inter-individual variance in warfarin dosing, respectively, with an increase in genetic polymorphisms found in Caucasian populations in comparison to African populations.^3^,^4^ Genetic polymorphisms are associated with decreased metabolism of or increased sensitivity to warfarin, as well as increased risk for bleeding events.^3^

There is a direct relationship between increased INR and risk of bleeding, with an INR > 4.0 associated with a high bleeding risk.^5^ Various risk factors predispose patients with therapeutic INRs to develop warfarin toxicity: dosing errors, drug–drug interactions, acute illnesses (diarrhoea, cardiac failure, hepatic impairment, fever) and dietary changes influencing vitamin K intake.^6^,^7^ Bleeding associated with warfarin toxicity carries a significant rate of morbidity and mortality. Risk factors for warfarin-associated bleeding mortality are advanced age, concomitant antiplatelet use, INR ≥ 4 at presentation, the use of vitamin K during hospitalisation, and intracerebral haemorrhage as a complication.^8^

Management of warfarin toxicity is determined by the degree of INR elevation with or without bleeding, and in the event of bleeding, the severity. Patients with an elevated INR and no evidence of bleeding can be managed with vitamin K, with or without omission of the next warfarin dose. Minor bleeding can be managed in a similar manner. The presence of major bleeding warrants immediate reversal of coagulopathy with the administration of vitamin K in conjunction with fresh frozen plasma (FFP) or 4-factor prothrombin complex concentrate (PCC). FFP and 4-factor PCC are considered to have a similar efficacy.^7^

There are no published data evaluating the causes, management and treatment costs of warfarin toxicity in South African healthcare facilities. The aim of this study was to provide an overview of warfarin toxicity, the management thereof and cost implications to treat a patient with warfarin toxicity in an academic hospital in South Africa.

## Methods

Ethical approval for the study was obtained from the Human Research Ethics Committee of the Faculty of Medicine and Health Sciences of the University of Stellenbosch (U15/09/002).

We conducted a retrospective review of adult patients (18 years or older) admitted to Tygerberg hospital (TBH), Cape Town, with warfarin toxicity during a one-year period from June 2014 to June 2015. Only patients known to be on established warfarin therapy were eligible for inclusion. Patients who were initiated on warfarin therapy during admission were excluded. Patients admitted more than once during the study period were recorded separately for each admission. We used National Health Laboratory Service (NHLS) records to identify patients with raised INRs and reviewed clinical notes, laboratory investigations and prescription data.

We collected demographic information, admission and discharge dates, INR measurements, the presence or absence of bleeding, sites and complications of bleeding, management, presumed cause of warfarin toxicity as recorded in the clinical notes, and whether it was addressed prior to discharge, as well as concomitant medicine use at time of admission. The cause of warfarin toxicity was recorded as not identified if a cause was not recorded in the clinical records. In the presence of bleeding, we classified it as major or non-major bleeding. Major bleeding was regarded as life- or limb-threatening bleeding, whereas all other cases where regarded as non-major bleeding.

We included patients presenting with warfarin toxicity, as defined by an admission INR value greater than 5. Patients included required at least one additional in-patient INR measurement to capture only in-patients. We excluded patients with an elevated INR who were not using warfarin and presented with elevated INRs due to other pathology such as liver impairment and sepsis. Patients with one elevated INR reading but who died prior to an additional INR measurement being done were not eligible for inclusion.

We calculated the warfarin toxicity treatment cost using the procurement cost of blood and blood products from the Western Cape Blood Transfusion Service, procurement cost of medicines from the TBH pharmacy, and cost to the hospital to admit a patient in a general ward in TBH using 2015 financial year costing. The general ward admission cost included personnel cost, clinical consumables, maintenance and engineering, equipment, services and overhead costs.

We used DRUG-REAX® Interactive Drug Interactions database (Truven Health Analytics Inc, Micromedex® Healthcare Series)^8^ to identify possible drug–drug interactions (DDI) between warfarin and drugs used by patients at the time of admission.

## Statistical analysis

No sample size was calculated and all patients identified during the study period were included. Data were entered into Microsoft Excel® and analysed using Stata version 11.0 (StataCorp, College Station, TX, USA). We assessed the normality of the data visually and using the Shapiro–Wilk test. Normally distributed data are described using the mean and standard deviation (SD) while non-normally distributed data are described using median and interquartile ranges (IQR). We explored statistical significance using appropriate tests for categorical, normal numerical and non-normal numerical data.

## Results

**Fig. 1.**
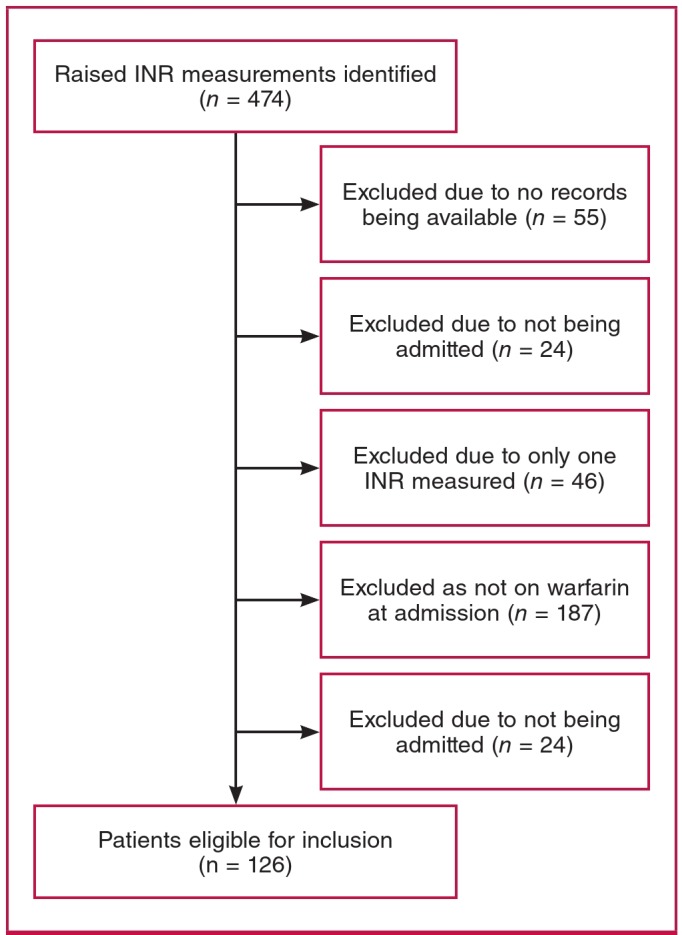
Study sample selection.

We identified 474 raised INR measurements (467 patients), of which 126 (122 patients) met our inclusion criteria for warfarintoxicity admissions (Fig. 1). Four patients presented with two admissions for warfarin toxicity during the study period and each admission was recorded. For clarity we will refer to the 126 warfarin-toxicity admissions as patients.

Sixty per cent (76/126) of patients were female and 40% (50/126) were male, with a median (IQR) age of 61 (48–70) years. Fifteen per cent (19/126) of patients died before discharge, although we could not attribute with certainty cause of death to warfarin toxicity. Patients were admitted for a median (IQR) of eight (5–16) days. The most common indications for the usage of warfarin were atrial fibrillation (AF) (57 patients), deep-vein thrombosis (DVT) (24 patients) and heart valve replacements (29 patients) (Table 1).

**Table 1:** Indications for warfarin therapy

*Indication*	*Number of patients*	*Percentage*
AF	48	38
Heart valve replacements	24	19
DVT	21	17
Other	21	17
Multiple indications (including, but not limited to AF, heart valve replacements and DVT)	9	7
Unknown	3	2
Total	126	

AF = atrial fibrillation, DVT = deep-vein thrombosis.

The median (IQR) admission INR was 8.49 (6.38–10) with the median (IQR) discharge INR 1.98 (1.28–2.82). The cause of warfarin toxicity was identified and addressed in 14.3% (18/126) of patients. Where the cause was identified, 55.6% (10/18) were due to dosing errors, 16.7% (3/18) DDIs, 11.1% (2/18) acute illnesses and 11.1% (2/18) due to inability to control INR despite best effort.

In cases of dosing errors, seven were due to physician error, two were due to patient error, and one was due to both physician and patient error. Physician error was due to a too-aggressive increase in warfarin dosage in response to previously sub-therapeutic episodes, and patient error was ascribed to incorrect and/or inconsistent usage of warfarin. In 85.7% of patients with warfarin toxicity, the cause was not identified (Table 2).

**Table 2:** Causes of warfarin toxicity

*Causes*	*No of patients*	*Percentage*
Cause identified	18	14.3
Dosing error	10	7.9
Physician	7	5.6
Patient	2	1.6
Both	1	0.8
Drug–drug interaction	3	2.4
Acute illness	2	1.6
Inability to control INR despite best effort	2	1.6
Other (liver injury)	1	0.8
Cause not identified	108	85.7
Total	126	

Eighty-five per cent (107/126) of patients were using concomitant medication on admission with 77% (97/126) of patients using one or more medicines with a known DDI with warfarin. The median (IQR) number of possible DDIs was two (one to three) per patient. The potential number of DDIs with warfarin per patient were: one DDI 18% (23/126), two DDIs 25% (31/126), three DDIs 18% (23/126), four DDIs 10% (13/126), five DDIs 4% (5/126), six DDIs 1% (1/126), and seven DDIs 1% (1/126). The most frequent drugs used found to have a DDI with warfarin were simvastatin (57 patients), aspirin (33 patients) and atenolol (29 patients). Table 3 reports on all major DDIs with warfarin.

**Table 3:** Major DDIs with warfarin

*Drug*	*Number of patients using drug*	*Quality of evidence of interaction*
Cardiovascular medicines		
Simvastatin	57	Excellent
Aspirin	33	Fair
Clopidogrel	4	Fair
Amiodarone	3	Excellent
Antimicrobial, including antiretroviral medicines		
Efavirenz	6	Fair
Amoxicillin	1	Good
Amoxicillin/clavulanic acid	1	Good
Ciprofloxacin	1	Good
Cotrimoxazole	1	Excellent
Moxifloxacin	1	Excellent
Metronidazole	1	Good
Central nervous system medicines		
Fluoxetine	4	Good
Citalopram	1	Good
Mirtazapine	1	Excellent
Valproic acid	1	Good

Twenty-eight per cent (35/126) of patients presented with major bleeding, 18% (23/126) with non-major bleeding and 54% (68/126) without bleeding. The most frequent sites of bleeding were upper gastrointestinal tract (31%, 18/58), haemoptysis (19%, 11/58) and epistaxis (17%, 10/58). Seven cases (12%, 7/58) of intracranial haemorrhage were reported. The median INRs for the major bleeding, non-major bleeding and non-bleeding groups were not significantly different (p = 0.05) at 10, 7.59 and 7.65, respectively.

We found no statistically significant relationship between the presence of DDIs and the occurrence of bleeding. Furthermore, although 36 patients were using concomitant antiplatelet medicines, no statistically significant relationships were found between the concomitant usage of antiplatelet medicines together with warfarin and the occurrence of bleeding (see Table 4).

**Table 4:** Bleeding versus antiplatelet medicines

*Bleeding*	*Aspirin*	*Clopidogrel*	*Aspirin and clopidogrel*
Major bleeding (n)	8	1	0
Non-major bleeding (n)	4	0	0
No bleeding (n)	20	2	1

The median number of treatment interventions was two, with 33.3% (42/126) of patients not receiving any interventions and 35.7% (45/126) and 23.8% (30/126) of patients receiving one and two treatment interventions, respectively. Nine (7.14%) patients received three or more interventions. Five per cent (6/126) of patients received three, 2% (2/126) received four and 1% (1/126) received five interventions, respectively.

The most frequently used interventions were vitamin K (45 patients), FFP (43 patients) and packed red blood cells (RBC) (34 patients). Factor PCC (Haemosolvex®) was administered in eight patients. Other interventions used were cryoprecipitate (one patient), tranexamic acid (two patients) and platelet products (three patients). See Table 5 for median (IQR) total dose given for the most frequently used interventions.

**Table 5:** Most frequent interventions given

*Intervention*	*Median total dose given (IQR)*
Vitamin K (oral/IV) (mg)	10 (5–20)
FFP (IU)	3 (2–4)
Packed RBC (IU)	2.5 (2–5)
4-factor PCC (IU)	1 250 (1 000–2 000)

IQR = interquartile range, IV = intravenous, FFP = fresh frozen plasma, RBC =red blood cells, IU = international units, PCC = prothrombin complex concentrate.

The average cost to treat a patient with warfarin toxicity was calculated at R10 578. The largest contributors to treatment costs were cost to be admitted and the use of blood and blood products when required (see Table 6).

**Table 6:** 

*Component*	*Cost average*	*Total range*
Hospital stay	R 7 464	(R 627 – R70 224)
Vitamin K	R 21	(R 1 – R 81)
FFP	R 3 948	(R 1 193 – R 10 737)
Packed RBC	R 4 617	(R 2 434 –R 15 821)
4-factor PCC	R 4 312	(R 1 568 – R 6 273)
Total cost to treat	R 10 578	(R 627 – R 79 762)

FFP = fresh frozen plasma, RBC = red blood cells, PCC = prothrombin complex concentrate.

## Discussion

To the best of our knowledge, this is the first review of warfarin toxicity in South Africa looking at causes, management and treatment cost implications. We found that the cause of warfarin toxicity was not identified in the majority of patients. DDIs were identified to be the cause of warfarin toxicity in only three cases, while we identified that 77% (97/126) of patients were using concomitant medication known to have a DDI with warfarin. The most frequently prescribed interacting medicines were cardiovascular medicines. Major DDIs with antimicrobial, antiretroviral and central nervous system medicines were also identified.

Our patients presented with significant morbidity, with nearly half of the patients presenting with bleeding, while 28% presented with major bleeding. Although many unrecorded DDIs with warfarin were identified, we could not prove a statistically significant relationship for the presence of DDIs and the occurrence of bleeding. Furthermore we could not prove a statistically significant relationship between the usage of antiplatelet medicines together with warfarin and the occurrence of bleeding.

Patients were admitted for a median of eight days and the average total cost to treat a patient with warfarin toxicity was estimated at R10 578. Of concern is that some patients were treated with high-cost interventions, which do not address the pathophysiology of warfarin toxicity. We also recognised a significant mortality rate associated with warfarin toxicity as 15% of patients died, although the final cause of death could not be attributed with certainty to warfarin toxicity.

The low pick-up rate for the cause of warfarin toxicity could be explained by physicians not documenting the cause of warfarin toxicity, however this is unlikely. Furthermore, it could be postulated that physicians are not aware of or unable to determine all the drug interactions with warfarin. Medicines found to have major DDIs with warfarin are used over a variety of disciplines and within a tertiary setting could result in the addition of medications to a patient’s treatment regime without adequate knowledge of already prescribed medication by other disciplines. Difficulty in dose adjustment could be explained by the availability of only 5-mg oral tablets in the public sector, limiting physicians in the degree that they can adjust warfarin dosage.

Our study has a number of limitations. Firstly, this was a retrospective study and relied on the availability of clinical records and the quality of available records. We were unable to obtain access to the clinical records of 55 raised INR measurements. Secondly, it is possible that we excluded patients presenting with warfarin toxicity complicated by major bleeding using our inclusion criteria. We identified 19 patients who died with only one INR measurement having been done, but who were excluded from our analysis due to insufficient clinical information and our inclusion criteria.

Thirdly, we were unable to determine the impact of genetics on warfarin toxicity. However, genotype-guided dosing is only of value when initiating warfarin therapy.^10^ Fourthly, INR measurements are reported up to 10 with values above 10 being reported as > 10 by the NHLS. For statistical analysis, values greater than 10 were processed as 10, and underestimated the association between INR and bleeding severity of warfarin toxicity. Lastly, we were not able to determine prolonged admission to hospital for concomitant medical or surgical conditions after correction of warfarin toxicity.

## Conclusion

We found that the cause of warfarin toxicity is frequently not identified by physicians and is therefore rarely addressed. We found that warfarin toxicity carries a significant morbidity rate and significant resources to treat. Future prospective research should study the causes of patients who are stable on warfarin treatment and present with warfarin toxicity, and target interventions to address this.
